# The impact of Teach-back method on preoperative anxiety and surgical cooperation in elderly patients undergoing outpatient ophthalmology surgery: A randomized clinical trial

**DOI:** 10.1097/MD.0000000000032931

**Published:** 2023-02-22

**Authors:** Dan Shen, Weiyi Huang, Shujin Wei, Yanjun Zhu, Baoxin Shi

**Affiliations:** a School of Nursing, Tianjin Medical University, Tianjin, China; b Outpatient Operating Room, Tianjin Eye Hospital, Tianjin, China.

**Keywords:** elderly patients, ophthalmic surgery, outpatient department, preoperative education, Teach-back

## Abstract

**Background:**

The literatures have demonstrated that Teach-back method is an effective communication tool to understand health education, especially in the elderly patients. However, there is limited research of Teach-back method in preoperative education for outpatient surgical patients. This study was conducted to investigate the effects of the Teach-back method on preoperative anxiety and surgical cooperation in elderly patients undergoing outpatient ophthalmology surgery.

**Methods:**

One hundred sixteen elderly patients who underwent outpatient ophthalmology surgery were selected as the research objects. They were divided into the observation group (58 cases) and the control group (58 cases). The Teach-back preoperative education was adopted in the observation group and the standard preoperative education method was adopted in the control group. The degree of anxiety, surgical cooperation, and awareness of health knowledge were compared between the 2 groups, and the variations of blood pressure and heart rate, as well as the highest values of intraoperative blood pressure and heart rate before and after method, were recorded and compared.

**Results:**

The preoperative systolic blood pressure in the observation group was significantly lower than that in the control group. The intraoperative (the highest value) heart rate, systolic blood pressure, and diastolic blood pressure in the observation group were lower than those in the control group, and the differences were statistically significant (*P* < .05). After intervention, the anxiety score and information demand score of the observation group were lower than those of the control group, and the differences were statistically significant (*P* < .05). The degree of surgery cooperation and awareness of perioperative health knowledge in the observation group were all higher than those in the control group; the differences were statistically significant (*P* < .05).

**Conclusion:**

The Teach-back method could relieve the preoperative anxiety of the patients, improve the quality of patients surgery cooperation, and facilitate the awareness of health knowledge. Moreover, it could effectively improve the intraoperative stress response of the elderly patients and reduce the large fluctuations of blood pressure and heart rate.

## 1. Introduction

Superficial ophthalmic surgery is generally conducted in the outpatient department of ophthalmology. Most of the surgeries could be completed under local anesthesia, the patients could also complete it with good cooperation. Unfortunately, the rapid surgery period, limited contact time between the nurses and patients, unfamiliar surgical environment, unknown surgical risks, and the lack of perioperative health knowledge would all contribute to enhancing the anxiety and fear of the patients. Moreover, the surgical operation, head position and eye position requirements, the stimulation of surgical pain in the awake state tended to cause psychological stress reaction, thus affecting the patients cooperation during the operation, and even influencing the surgical performance.^[1]^ Therefore, it is of great significance for the patients to fully understand the whole process of outpatient surgery through comprehensive preoperative education.^[2]^

Currently, various forms of preoperative education have been reported,^[3,4]^ most of them were in the form of indoctrination. However, the cognitive function of the elderly patients would be impaired with age, and it was difficult to fully grasp the contents of method in a short period of time. Most elderly patients are unaware of their own lack of knowledge. The Teach-back method was simple and easy to operate, and this method has also been widely employed in the health education of the elderly patients worldwide to improve the health behaviors and self-management ability of the elderly patients.^[5]^ Nevertheless, its application performance in outpatient and preoperative education was rarely reported. In the present study, the superiority of Teach-back method was verified by comparing the application effect of Teach-back method and traditional 1-way communication method on elderly patients in outpatient ophthalmic surgery.

## 2. Materials and methods

### 2.1. General materials

**Figure 1:**
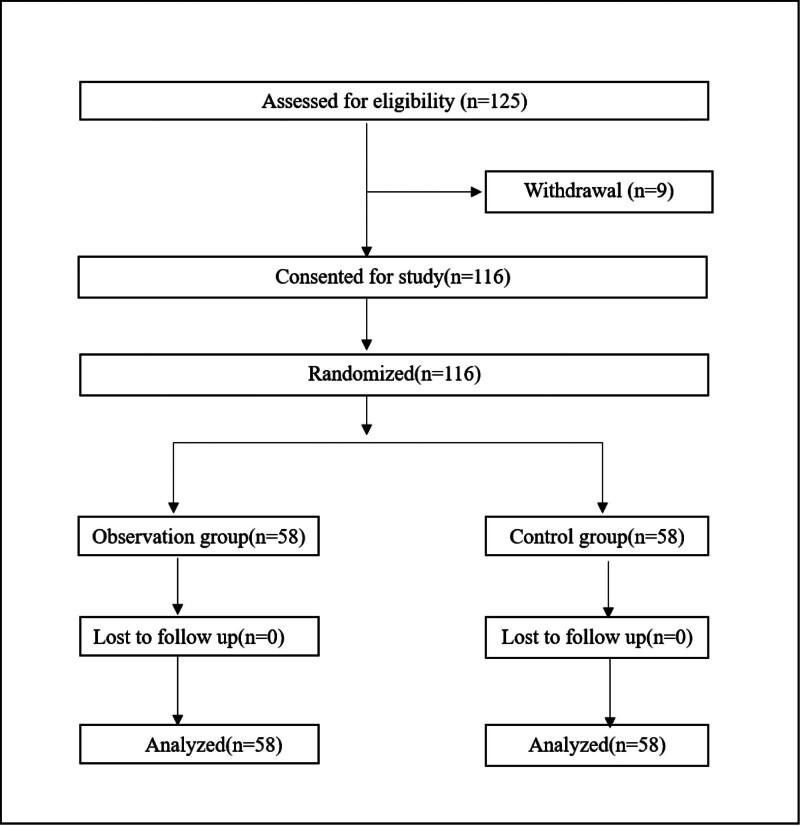
CONSORT diagram showing the flow of participant recruitment throughout each stage of the randomized trial. Patients in group S1 adopted Teach-back preoperative education, the patients in group S2 adopted the standard preoperative education. CONSORT = Consolidated Standards of Reporting Trials.

The research was approved by Ethics Committee of Tianjin Eye Hospital. The study was registered in the Chinese clinical trial registry under the number ChiCTR2200061911 and first registration date July 11, 2022. The elderly patients who were scheduled to undergo surgery in the outpatient department of Tianjin Eye Hospital from July 2022 to September 2022, were selected as the research objects. And the objects were divided into 2 groups by random number table method, including 58 cases in the observation group and 58 cases in the control group. All the study objects had informed consent and volunteered to participate in the study. Inclusion criteria: American Society of Anesthesiologists grade: I to II; Age ≥60 years old; Clear consciousness, barrier-free language communication, and basic listening, speaking, reading, and writing skills; Receiving ophthalmic local anesthesia surgery for the first time; Patients who were volunteered to participate in the group. Exclusion criteria: Severe systemic complications; Severe cognitive impairment or mental illness. Elimination criteria: Voluntary cancelation of the surgery and noncooperation with follow-up. The patient asked to withdraw from the study. The flow chart of the process of subject inclusion is presented in Figure 1.

### 2.2. Intervention methods of observation group

#### 2.2.1. Establish a Teach-back method group.

The team members included 4 operating room nurses and 1 deputy chief doctor. The doctor is in charge of reviewing method content and providing professional support, the nurse is responsible for the implementation of preoperative education. The nurses have more than 5 years of the related work experience and good communication skills, learn the implementation steps and precautions of Teach-back method through online video training, literature reading, etc, and pass the simulation testing in pairs.

#### 2.2.2. Formulate a Teach-back preoperative education evaluation scale.

Organized to discuss the content of perioperative education, and formulated a Teach-back perioperative education evaluation form: Before the surgery, it included understanding the basic situation of the patients, previous illness history and medication history, and emphasizing the importance of blood pressure and blood glucose control; Inform the patients of the tips such as surgical methods, dressing requirements, preoperative diet guidance; Cooperation method during the surgery: explained the purpose, content and importance of eye position fixation training, the way of communication with doctors and the way of relaxation; Postoperative drug administration method and review time. Ensured that the members were proficient in perioperative knowledge and could comprehensively answer and address the questions raised by the patients.

#### 2.2.3. Specific implementation process of Teach-back.

Distributed preoperative education manuals before the surgery, explained to the patients in different periods according to the method plans, demonstrated to the patients the methods of fixation training, the techniques of relieving cough or sneeze during the surgery, and the specific methods of drug administration after the surgery; At the end of a section of education, you could ask the patient questions, such as “We have understood the precautions before surgery, please review the content with me in your own words.,” “In order to make sure that I explained it clearly, please show me how you should do when the doctor says ‘look left’ during the surgery?.” Asked the patient to demonstrate the cooperation method or describe the education contents in their own words. The team members assessed the patients understanding and mastery of the education information according to the Teach-back method assessment scale; Clarified and corrected all the information that the patient had misunderstood and comprehended incompletely. Used a kind voice instead of an accusatory and interrogative tone to avoid the patients discomfort due to insufficient understanding. Stages 2 and 3 could be repeated continually until the patients understood all the information; If the patient still could not recall the information correctly after 3 times of repetition, considered the help of their family members or used other tools to repeat the explanation again; if the patients did not accept this method, it should be replaced with an easy-acceptable method.

#### 2.2.4. Control group.

The patients were given standard method: Preoperative distribution of health education manuals, including surgery time, preoperative examination, preoperative diet, dress requirements and other precautions, and to understand the medication status of the patients basic diseases; Informed the patients of the surgery cooperation method: explained the eye position fixation method, the communication method with the doctor and the relaxation method during the surgery; Explained the method of drug administration and review time after surgery. Explained and answered the patients questions after the education.

### 2.3. Observation indicators

#### 2.3.1. General information questionnaire.

The questionnaire was designed by the researchers, including demographic data (gender, age, education level, et al) and disease surgical data (diagnosis, surgical methods).

#### 2.3.2. Heart rate and blood pressure.

Measured and recorded the patients systolic blood pressure, diastolic blood pressure, heart rate before intervention and surgery, as well as the highest values of systolic blood pressure, diastolic blood pressure, and heart rate during the surgery.

#### 2.3.3. Amsterdam Preoperative Anxiety and Information Scale (APAIS).

The APAIS scale^[6]^ has been translated into many languages since the APAIS Dutch version was published in 1996. The Chinese version of the APAIS scale was translated into Chinese by Wu Hao et al in 2016,^[7]^ and possessed good reliability and validity. It was easy to use and had good correlation, and the patients could complete the scale within 2 minutes. Items 1, 2, 4, and 5 were preoperative anxiety scores. The higher the score was, the higher the level of anxiety would be. Items 3 and 6 were the information demand scores, with a score of 2 to 4 indicating low information demand, 5 to 7 indicating moderate information demand, and 8 to 10 indicating high information demand. The Cronbach α coefficient of the Chinese version of the APAIS scale was 0.832, and the Cronbach α coefficient of the anxiety scale and the information needs scale were 0.840 and 0.782, respectively.

#### 2.3.4. Evaluation of surgical cooperation degree.

According to the literature^[8]^ and clinical experience, the self-formulated evaluation scale of the patients surgical cooperation degree was used. The scale was divided into 3 grades according to the cooperation degree: good, general, and poor. I-Good: the patient head could remain still during the surgery, and the eye position and fixation could be changed according to the doctor instructions; II-General: During the operation, the inclines of the patient head caused the deviation of the surgical field, or failing to rotate the eyeball caused the deviation of the eye position. The medical staff reminded the patient to adjust the position <3 times; III-Poor: deviation of the surgical field or eye position more than 3 times, or touched the contaminated sterile area.

#### 2.3.5. Perioperative health knowledge awareness questionnaire.

On the basis of references,^[9,10]^ the questionnaire was designed by ourselves. The contents of the questionnaire included 3 dimensions of preoperative precautions, intraoperative coping and postoperative precautions. The contents contained 15 items, with each item being divided into 4 grades “know completely, know most of the contents, know a little, don’t know,” corresponding to the scores of “4, 3, 2, and 1” respectively. The full score of the questionnaire was 60 points, ≥48 points indicated knowing, 36 to 48 points indicated basically know, and <36 points indicated don’t know. The Cronbach α coefficient of the scale was 0.872.

### 2.4. Statistical method using SPSS 26.0 statistical software

Through a pilot study (unpublished), we obtained the control group mean APAIS anxiety score (7.24) and standard deviation value (2.2). And it is expected that the intervention group will reduce the anxiety score by 2.4 points. At α = 0.05 and β = 0.1, the sample size was calculated by the sample size calculation formula of 2-group mean comparison was 46 cases, and the sample size of each group was 56, based on the 20% shedding rate of each group.

The measurement data were expressed as $\bar{x}$ ± s, the variance analysis of repeated measurement data was used for intragroup comparison, and the LSD method was used for intra group comparison. *T* test of independent samples was employed for between-group comparisons. The counting data were expressed by frequency and percentage, using $\chi^{2}$ tests, the rank sum test was used for grade data, and *P* < .05 indicated that the differences were statistically significant. In this study, we adopted the APAIS anxiety score as the main observation end point.

## 3. Results

### 3.1. Comparison of general data between the 2 groups

There was no significant difference in gender, age, education level and type of surgery between the 2 groups (*P* > .05). See Table 1 for the details.

**Table 1:** Comparison of general data between the 2 groups.

	Observation group, n = 58	Control group, n = 58	Test statistics	*P* value
Age	67.10 ± 5.02	66.14 ± 4.59	1.081	.282
Gender				
Male	28	32	0.552	.577
Female	30	26		
Education level				
Below senior high school	36	33	0.322	.705
Above senior high school	22	25		
Surgery				
Vitreous injection	7	8	0.608	.895
Types				
Pterygium combined with autologous limbal epithelial transplantation	24	20		
Cataract phacoemulsification combined with intraocular lens implantation	13	15		
Excision of conjunctival tumor	14	15		

### 3.2. Comparison of blood pressure and heart rate at different time points between the 2 groups

There was no significant difference in systolic pressure, diastolic pressure and heart rate between the 2 groups before intervention (*P* > .05); After intervention, the systolic blood pressure in the observation group was significantly lower than that in the control group before the surgery, with a statistically significant difference (*P* < .05); The systolic blood pressure, diastolic blood pressure and heart rate of the patients in the observation group were lower than those in the control group during the operation (the highest value was taken), and the differences were statistically significant (*P* < .05). See Table 2.

**Table 2:** Comparison of blood pressure and heart rate between the 2 groups.

Measurement time point	Hemodynamic indexes	Observation group (n = 58)	Control group (n = 58)	*t* value	*P* value
Before intervention	Systolic pressure	142.2 ± 6.5	142.3 ± 7.7	−0.047	.963
	Diastolic pressure	85.6 ± 4.8	87.0 ± 6.2	−1.403	.163
	Heart rate	72.2 ± 5.0	70.8 ± 5.3	1.486	.140
Preoperative after intervention	Systolic pressure	148.1 ± 7.0	151.5 ± 8.1	−2.403	.018
	Diastolic pressure	87.9 ± 6.8	89.4 ± 6.7	−1.206	.230
	Heart rate	73.6 ± 6.6	73.6 ± 5.6	−0.038	.970
During the surgery (the highest value was taken)	Systolic pressure	154.2 ± 8.3	160.0 ± 7.8	−3.921	.000
	Diastolic pressure	87.1 ± 6.9	92.3 ± 7.3	−3.974	.000
	Heart rate	76.3 ± 5.6	83.0 ± 6.2	−6.071	.000

### 3.3. Comparison of APAIS scores between the 2 groups before and after intervention

There was no statistically significant difference in APAIS scores between the 2 groups before intervention (*P* > .05). After intervention, the anxiety scores and information demand scores of the observation group were lower than those of the control group, and the differences were statistically significant (*P* < .05). See Table 3.

**Table 3:** Comparison of APAIS scores between the 2 groups before and after intervention (points, $\bar{x}$ ±s).

Items	n	SumS		SumI	
		Before intervention	After intervention	Before intervention	After intervention
Observation group	58	7.24 ± 2.20	4.78 ± 1.23	4.34 ± 1.82	2.53 ± 0.82
Control group	58	7.22 ± 2.23	5.50 ± 1.45	4.17 ± 1.53	2.93 ± 1.10
*t* value		0.042	−2.897	0.551	−2.192
*P* value		.967	.005	.583	.030

APAIS = Amsterdam Preoperative Anxiety and Information Scale.

### 3.4. Comparison of the degree of surgical cooperation between the 2 groups

The rank sum test was used to compare the 2 groups, and the differences were statistically significant (*P* < .05). See Table 4 for details.

**Table 4:** Comparison of the degree of surgical cooperation between the 2 groups.

Items	n	Degree of surgical cooperation		
		Good	General	Poor
Observation group	58	33	20	5
Control group	58	22	27	9
*Z* value		−2.076		
*P* value		.038		

### 3.5. Comparison of the mastery of perioperative health knowledge between the 2 groups

The total score of perioperative health knowledge in the observation group was higher than that in the control group, and the differences were statistically significant (*P* < .05). The awareness of perioperative health knowledge in the observation group was higher than that in the control group, the differences were statistically significant (*P* < .05). See Table 5.

**Table 5:** Comparison of scores and awareness of health education knowledge between the 2 groups.

Items	n	Total scores ($\bar{x}$ ± s)	Awareness		
			Know	Basically know	Don’t know
Observation group	58	53.19 ± 6.42	36	22	0
Control group	58	47.45 ± 8.10	24	26	8
*t*/*Z* value		4.228	−2.708		
*P* value		.000	.007		

## 4. Discussion

The Teach-back communication strategy has been applied in many fields of clinical areas,^[11]^ including chronic disease management, surgical rehabilitation training and discharge information comprehension. It was a patient-oriented, dynamic and interactive communication method, which could facilitate the patients understanding and satisfaction. Compared with the traditional 1-way instillation education method, the Teach-back method focused more on the patients understanding of health knowledge, and the understanding of the patients and whether the patient really grasped the information conveyed by the medical staff. It would be beneficial to conduct timely evaluation, feedback, and targeted re-guidance, which would be helpful to form a correct understanding of diseases and surgeries, and improve the effect of education implementation. Meanwhile, repeated and patient communication would help optimize the relationship between the nurses and patients, improve the trust of medical staff, increase the patients confidence in surgery and relieve the tension and anxiety of the patients.^[12]^ Elderly patients were more prone to generate anxiety and panic psychology because of the aging of body tissues and organs, the decline of regulatory function, and slow information reception, insufficient understanding of health education content.^[13,14]^ Moreover, it would also induce the variations such as increased blood pressure and heart rate, and thereby increasing the potential risk of surgery.^[15]^

In the present study, the preoperative anxiety and information demand of the patients who used Teach-back method have significantly improved. After the intervention, the preoperative systolic blood pressure, and the highest value of intraoperative blood pressure and heart rate of the patients in the observation group were significantly lower than those in the control group. It might be attributed to the reason that the Teach-back method enabled the patients to grasp more comprehensive information about the surgery, reduce anxiety, and thus alleviating the cardiovascular reactions caused by tension.^[16]^

It has been reported that up to 40% to 80% of medical information would be immediately forgotten during the method process, many recalled information would be erroneously retained.^[17]^ The Teach-back educational method had a basic theory in cognitive psychology, indicating that the repeated short sequences of information would be beneficial to improve the recall of information, and was an effective intervention to improve information memory and understanding.^[18]^ Xia J et al^[19]^ reported that the Teach-back education method could effectively improve the indicators of diabetes patients such as treatment compliance and life quality. Ophthalmic surgery was a delicate operation that required a high degree of patients cooperation. A full understanding of the surgical process would help the patient better cooperate with the doctor to complete the surgery. Alternatively, inappropriate or even wrong communication methods and fixation methods during the operation would not only affect the degree of surgical cooperation, but also lead to adverse consequences including infection and corneal damage.^[20]^

The results indicated that the patients in the observation group had a higher degree of surgical cooperation than those in the observation group. The open feedback of Teach-back method would contribute to increasing the patients sense of participation and improving the completion of surgery-related cooperation training. Mahajan M et al^[21]^ pointed out that the proportion of the patients with comprehension deficits decreased from 49% to 11.9% when the Teach-back education model was employed. And it was also found that the Teach-back method could effectively improve short-term recall and understanding ability of the patients. Moreover, White M et al^[22]^ demonstrated that the Teach-back model could improve the retention rate of health knowledge in elderly patients with heart failure (≥65 years old). This was consistent with the results that the awareness of perioperative health knowledge of patients in the observation group was higher than that of the patients in the control group. The understanding ability and memory of elderly patients would decline with age. Through the process of self-reported feedback, the elderly patients could not only deepen their memory impression, but also reflect their mastery of health knowledge. Forgotten and misunderstood education information can be repeatedly supported and guided by information, thereby improving the method effect.

Teach-back method is a 2-way information transmission mode, the core of it is to explain, and supplement and strengthen the understanding of the contents according to the feedback of the patients on the mastery of the education contents. The method is simple and time-saving, ensures that the patients fully understand and master the contents of the education in a short period of time.^[23,24]^ The application results of this study in outpatient ophthalmic surgery of elderly patients indicated that the Teach-back method could effectively improve the preoperative stress response of the elderly patients, reduce the large fluctuations in blood pressure and heart rate, relieve the patients preoperative anxiety, improve the quality of the patients surgical cooperation, and as well as raise the awareness of health knowledge. However, this study has certain limitations because the investigation was conducted in only 1 hospital. In the future, the reliability of the research results can be further verified by expanding the sample size or the combination of multiple centers.

## Author contributions

**Conceptualization:** Dan Shen, Baoxin Shi.

**Data curation:** Dan Shen, Weiyi Huang, Shujin Wei, Yanjun Zhu.

**Formal analysis:** Dan Shen, Shujin Wei, Yanjun Zhu.

**Investigation:** Weiyi Huang, Yanjun Zhu.

**Methodology:** Dan Shen, Shujin Wei, Baoxin Shi.

**Project administration:** Shujin Wei, Baoxin Shi.

**Resources:** Weiyi Huang, Baoxin Shi.

**Software:** Shujin Wei, Baoxin Shi.

**Supervision:** Shujin Wei, Baoxin Shi.

**Validation:** Weiyi Huang, Baoxin Shi.

**Visualization:** Weiyi Huang, Baoxin Shi.

**Writing – original draft:** Dan Shen.

**Writing – review & editing:** Shujin Wei, Baoxin Shi.
